# Laboratory Evaluation of Storage Bags for Infestations in Wheat Caused by *Rhyzopertha dominica* F. (Coleoptera: Bostrichidae) and *Trogoderma granarium* Everts (Coleoptera: Dermestidae) and Their Control Using Phosphine Fumigation

**DOI:** 10.3390/insects13100955

**Published:** 2022-10-19

**Authors:** Hafiz Waqas Waheed, Muhammad Waqar Hassan, Ghulam Sarwar, Moazzam Jamil

**Affiliations:** 1 Department of Entomology, Faculty of Agriculture and Environment, Islamia University of Bahawalpur, Bahawalpur 63100, Pakistan; 2 Department of Botany, Faculty of Chemical and Biological Sciences, Islamia University of Bahawalpur, Bahawalpur 63100, Pakistan; 3 Department of Soil Science, Faculty of Agriculture and Environment, Islamia University of Bahawalpur, Bahawalpur 63100, Pakistan

**Keywords:** cereals, grains, economic loss, control, integrated management, phosphine fumigation

## Abstract

**Simple Summary:**

Wheat is commonly stored in bags in Asian countries, which include jute bags, woven plastic bags, and hermetic plastic bags. We evaluated these bags under laboratory conditions for external infestations caused by two serious pests of wheat. Considering all three bags, results showed that insects preferred to enter jute bags due to their loose fibers (termed as invasions), while no insects created holes or entered (penetrations) in woven plastic bags and hermetic plastic bags which had no such openings. In the second experiment, insects were released in jars with only two types of bags, namely woven plastic bags and hermetic plastic bags. In this case, insects created holes and penetrated into woven plastic bags but not into hermetic plastic bags. Both these experiments showed hermetic plastic bags remained safe from external infestations due to their thick and smooth plastic. The third experiment evaluated fumigation efficacy against all three bags for the pests already present in bags with grains. Results showed mortality was maximum in jute and woven plastic bags but negligible in hermetic plastic bags. The results of these experiments pinpointed deficiencies in all three bags for controlling insect infestations (external or internal) and demand alterations in these bags to successfully control such infestations.

**Abstract:**

Bag storage of wheat is common in Asian countries, and common types of such bags include jute bags, woven plastic bags, and hermetic plastic bags. In order to assess infestation by two serious pests, namely *Rhyzopertha dominica* (Coleoptera: Bostrichidae) and *Trogoderma granarium* (Coleoptera: Dermestidae) in these bags, two experiments were performed. In the first experiment, three popular wheat varieties, namely Akbar, Dilkash, and Bakhar star were filled in above-mentioned miniature-size bags which were then placed in jars with three replicates per bag type and variety. Forty insects (adults for *R. dominica* and larvae for *T. granarium*) were released in the center of the jars for a period of 30 d in two different setups for both species. Data were recorded twice: after 15 d and 30 d. Results showed insects entered the jute bags only (made invasions due to its loose fibers and openings). Inside the bags, *R. dominica* caused more weight loss and live insects in Dilkash and Akbar varieties, while *T. granarium* preferred Bakhar star and Dilkash compared with their third variety. In the second experiment, only two bags, namely woven plastic bags and hermetic plastic bags filled with one variety, were tested to check damage (penetrations because no openings are present in these bags as in jute bags) to the bags. Results showed both species created holes in woven plastic bags but not in hermetic plastic bags. These results showed hermetic plastic bags remained safe from external infestation compared with the other two bags. Sometimes if insects are already present in the grains inside the bags, fumigation is needed from outside to kill the pests inside. To evaluate this, all three bags were filled with a wheat variety and were also infested with both insect species and placed in a fumigation container with nine replicates per bag type. A phosphine tablet (3.0 g) wrapped in muslin cloth was placed in a container which was then sealed, and the fumigation-induced mortality after 24 h was recorded. Results showed mortality was >95% to 100% in woven and jute bags, respectively, while mortality in hermetic plastic bags remained very low (<3%). These results revealed the least fumigant gas permeation in hermetic plastic bags compared with jute and woven plastic bags. Results of all three experiments demand immediate alteration in creation of all three bags to curtail infestation from outside (jute and woven plastic bags) as well as to generate maximum fumigation efficacy when the source of infestation is with the grains (hermetic bags).

## 1. Introduction

Cereal grain crops provide more food energy worldwide than any other types of crops and are, therefore, staple crops. Among cereals, wheat (*Triticum aestivum* L.) is one of the most extensively grown crops in the world, cultivated on more than 250 million hectares globally [1], which is larger than for any other crop; furthermore, world trade is greater for wheat than for all other crops combined. Postharvest losses are often reported due to inadequate and inefficient storage facilities, which may be due to problems arising from different biotic and abiotic factors. Storage insect pests, which attack the stored cereals grains and destroy their quantity as well as their quality, constitute a serious threat among the biotic factors. If even a single insect gets into the packing, there is a good chance it will cause a lot of damage.

It has been reported that 7 out of 18 listed stored grain insect pests as the most destructive species in Punjab, Pakistan. These include *Trogoderma granarium*, *Rhyzopertha dominica*, *Tribolium castaneum, Sitotroga cerealella*, *Sitophilus oryzae*, *Corcyra cephalonica*, and *Callosobruchus chinensis* [2]. Their presence in food and durable commodities can have both direct and indirect effects on human health [3]. Khapra beetle *Trogoderma granarium* is one of the most notorious pests of stored grains and is regarded as a major threat to food security across the world [4]. Being a primary pest, it consumes the whole grains, leaves the husk, and decreases seed germination ability [5,6]. It can increase its population rapidly under favorable conditions. During unfavorable conditions, this insect can enter larval diapause and can survive in this state for several years. This pest mostly spreads through commercial trade. Adults are in the non-feeding stage, while major damage to grains is caused by their larvae. *R. dominica* is another primary pest of stored grains in many parts of the world [7]. It causes damage to stored cereals such as wheat, rice, corn, sorghum, tubers, starch-containing substrates, and packaging made from wood. They not only cause quantitative losses, but they also have an impact on the quality of grains during the storage period due to their feeding activities [8]. Unlike *T. granarium*, both larvae and adults of *R. dominica* cause damage to wheat. Their larvae are essentially internal feeders but can also develop as external feeders of stored grains. Usually, their larvae and pupae remain hidden during immature stages within grains, which make them difficult to spot, and, therefore, management of such pests deserve further efforts.

In developing countries such as Pakistan, large-scale bag storage of wheat includes usage of different storage bag types such as jute bags, woven plastic bags, and hermetic plastic bags. The bag storage of wheat is so common that there are separate industries for manufacturing of these storage bags; losses in grains are also huge irrespective of the type of storage bags being used to store these grains. Due to losses reported, pesticides are routinely sprayed on bag surfaces inside wheat warehouses to protect grains from insect attack. Different authors have emphasized the importance of pesticide application on different types of storage bags surfaces to control insect pests [9]. However, restrictions on pesticide use and emphasis on hygiene may be hindered to some extent by strict production schedules, so the development of insect-resistant packaging is increasingly important for both consumers and manufacturers [10]. Indiscriminate use of insecticides directly on commodities for direct human consumption is hardly acceptable. There are also growing concerns about environmental pollution and development of insecticide resistance in storage insect pests due to improper use of these liquid chemicals.

One of the best options as an alternative to grain protectants is fumigation with phosphine, which has no harmful effects on environment and leaves no residues in commodity. It has excellent penetration ability to kill internal pests or those present deep inside grain mass. It is less expensive, easy to apply in warehouses or in airtight containers, and is readily available in the market. In countries where mixing of chemicals with grains is prohibited, phosphine fumigation serves the best purpose to kill and control storage insect pests in grains [11].

Our earlier research for management of important storage pests in packaged foodstuffs included evaluation of some frequently used plastic packing for their resistance to external insect infestation in these bags and their efficacy for the vital fumigant (phosphine) gas permeation if infestation was already present in foodstuffs; this included evaluation of polyethylene, polypropylene, and polyvinylchloride packaging against different insect pests, namely *Tribolium castaneum*, *T. granarium*, and *R. dominica* [12,13,14,15,16].

There are regular reports of grain losses in all types of storage bags caused by different types of storage pests. However, research work on causes of pest infestation from outside these bags and pest control in these bags with fumigation if infestation is already there is limited. Therefore, it is reasonable to evaluate the frequently used bags, namely jute bags, woven plastic bags, and hermetic plastic bags for their ability to resist insect infestation from outside (invasions or penetrations) against *T. granarium* and *R. dominica*. Secondly, if these pests are already present in the grains, all three bags shall also be evaluated for their efficacy for phosphine gas permeation from outside in the form of mortality of these pests. Inclusion of three popular wheat varieties, namely Akbar, Bhakar star, and Dilkash in these bags is evaluated alongside bag types, and the effect of these varieties on dependent variables, namely live insects inside bags and percent weight loss due to these pests in wheat, is investigated.

## 2. Materials and Methods

### 2.1. Collection and Rearing of Insects

Populations of *Rhyzopertha dominica* and *Trogoderma granarium* (larvae and adults) were collected from different locations in Bahawalpur, Punjab, Pakistan. The insects of both species were reared separately to form a single colony of each species in the stored grains insect pests rearing section of the Department of Entomology, Faculty of Agriculture and Environment (FA & E), The Islamia University of Bahawalpur, Pakistan. Laboratory-maintained collections are available from ten years in the Department and are augmented with new populations when needed.

Both species were reared in clear plastic jars of 0.5 liter volume capacity. Whole wheat grains were used as rearing media for both species. Jars were covered with muslin cloth with the help of a rubber band to prevent the beetles from escaping from jars and also for ventilation. Adults of *T. granarium* and *R. dominica* were released (50 male and 50 female) into the rearing jars to get a homogenous population. The plastic jars were kept in the laboratory rearing room under controlled conditions at 30 ± 2 °C and 65 ± 2% RH. Data on relative humidity and daily temperature were recorded by a thermo-hygrometer.

### 2.2. Wheat Storage Bags and Wheat Varieties

Three types of frequently utilized wheat storage bags, namely jute bags, woven plastic bags, and hermetic plastic bags (HAJI SONS, Lahore, Pakistan), were purchased from the wholesale market in Lahore, Pakistan. In the laboratory, small bags of these wheat storage bag (8 × 12 cm) were prepared with the help of an impulse sealer by a heat-sealing process for the hermetic plastic bags, while woven plastic bags and jute bags were prepared of the same size by means of smart needlework performed by machine to ensure that no external vents were there due to sewing. In addition to storage bags, to see the effect of wheat varieties on dependent variables, three prominent varieties of wheat, namely Akbar, Dilkash, and Bhakkar Star, were obtained for research purpose from Regional Agriculture Research Institute (RARI) Bahawalpur, Pakistan which works under the umbrella of Ayub Agriculture Research Institute (AARI) Faisalabad, Pakistan. To kill any prior insect infestations (eggs, larvae, pupae), the grains so obtained were heated in an oven at 60 °C for 15 min after which these were stored in separate vessels along with variety tags.

### 2.3. Experimental Setup (Choice Test between Invasions and Penetrations)

Before the start of experiments, grains of three wheat varieties were spread in trays separately in a single file for several days to normalize moisture contents in grains with the laboratory conditions. On the day of the experiment, grains of all three varieties were weighed using sensitive electrical weighing balance, and measured quantities of only sound grains were used in experiments. Three types of storage bags as described above were filled with wheat grains (60 g each for *T. granarium* and *R. dominica*) of one variety and their mouths through which grains were filled were sealed either by heat sealing for hermetic plastic bags or were stitched by fine needle work from upside in the case of woven plastic bags and jute bags. A plastic jar (1.0 L volume capacity) into which three types of bags with one wheat variety were placed was positioned vertically along a wall. Then, 40 adults of *R. dominica* were released in the center of this experimental jar outside of the bags. These three types of bags with one variety were replicated thrice in three such jars followed by the release of insects in them to make three replications for each treatment, namely bag types and wheat variety. Similarly, the other two varieties were filled in these three types of bags in same manner and placed in jars followed by release of the insects. Similar experimental setup was repeated for *T. granarium* as well in which 3rd to 4th instars larvae were released in the center of jars. Data were recorded after 15 and 30 days post experiment initiation. After 15 days, jars were inspected to record data. At first, dead insects outside the bags were counted in each jar. Bags were inspected from outside for damage such as holes. In cases of no external damage to the bags, these were counted as having no live insects or percent weight loss in them due to no insects inside. Afterward, bags were opened to record the number of insects which entered the bags. Later, damaged grains were separated from uninfected grains and were counted and weighed. Similarly, undamaged grains were also counted and weighed.

### 2.4. Weight Loss Assessment

Inside bags with insects, data regarding numbers of damaged grains, their weight, numbers of undamaged grains, and their weight were recorded in each treatment per replicate. Data were used to calculate percent weight loss with respect to each treatment using the formula by [17].

(1) $$\mathrm{Percent}\;\mathrm{weight}\;\mathrm{loss}=\frac{\left(Wudg\times Ndg\right)-\left(Wdg\times Nudg\right)}{Wudg\times \left(Ndg+Nudg\right)}\times 100$$

where *Wudg* = weight of undamaged grains, *Nudg* = number of undamaged grains, *Wdg* = weight of damaged grains, and *Ndg* = number of damaged grains.

Following data collection, healthy grains were repacked into their respective bags, number of damaged grains were replenished with sound grains to restore the original number of grains, the originally released insects outside the bags were restored by releasing new insects, and the bags were resealed using the impulse sealer for hermetic plastic bag types and restitched in the case of woven plastic bags and jute bags. Following this setup, new data of the same parameters were recorded 30 days post initial setup.

To distinguish between feeding habits of two species, per grain damage caused by both insects was calculated. This was done by totaling the weight of all damaged grains by *T. granarium* and *R. dominica* per treatment divided by numbers of damaged grains by these species. Thus, average weight of a damaged grain was obtained in three varieties for *T. granarium* and *R. dominica*.

### 2.5. Penetration Test

The bags which did not show any symptoms of attack by insects in the first experiment, namely woven plastic bag and hermetic plastic bag, were evaluated further for penetration test in the second experiment in terms of holes and insect penetrations. For this purpose, bags of hermetic and woven plastic were prepared similarly to the previous experiment and placed inside a plastic jar of 1.0 L volume capacity in an upright position. In this way, each bag type was filled with the preferred wheat variety for each insect type, as described in the first experiment (Bakhar star for *T. granarium* and Dilkash for *R. dominica*). Nine such plastic jars, with two such bags one each for hermetic and woven plastic served as nine replicates, were arranged in a randomized complete design for each insect. This was then followed by the release of 50 3rd and 4th instar larvae of *T. granarium* in the center of each jar outside the bags. This setup was retained for a period of ten days to record holes in bags created by insects. If the bags had holes, these were opened to record (penetrations) of the insects which entered through holes in these bags. The same set up and experiment procedure was repeated for *R. dominica* in which 50 *R. dominica* adults were released outside the bags inside jars.

### 2.6. Fumigation Test

In order to investigate the effect of bag types, all three bags were evaluated in the third experiment for phosphine fumigation efficacy. For this purpose, all three bag types were prepared similarly to the first experiment and filled with one wheat variety (as in the second experiment), with nine replicates for three bag type. These bags were then infested with 10 live larvae of *T. granarium* (3rd to 4th instars) from the culture. Following that, all bags containing wheat grains and insects were sealed accordingly, as in first experiment. In this set up, control treatments were also prepared which comprised similar bag types containing the wheat as described above and infested with similar number of insects but without fumigant application. In this way, at first fumigation, treatment bags were placed in a square-shaped fumigation tin container of 0.7 m cube volume capacity (in order to achieve the fumigation dose of phosphine @ 1.5 g (gas)/m) followed by placing of one (3.0 g) tablet of aluminum phosphide ((56 % *w/w*) ICI Pakistan Ltd.) because a 3.0 g tablet releases 1.0 g gas on contact with ambient air. The bags were arranged in the bottom of container to represent nine replicates per treatment. One aluminum phosphide tablet wrapped in muslin cloth was placed on the center top location in container. Following this experimental setup, the lid of the tin container was closed and wrapped tightly with broad packing tape to ensure airtight conditions for maximum fumigation efficacy. Control treatments included repeating the same experimental setup for all replicates and in a similar manner as described above in the tin container but without the phosphine tablet application. Mortality of insects in treatments and in control (if any) was recorded 24 h post treatment period. The same setup was repeated for *R. dominca* regarding bag types and fumigation application in which bags were infested with 10 live adults of *R. dominica*.

### 2.7. Statistical Analysis

Data were analyzed separately for both insects using IBM SPSS Statistics (Version 28) [18] Data for first experiment were analyzed by GLM-repeated measures ANOVA in which the treatment variables were the three types of storage bags and three wheat varieties, and the dependent variables were live insects inside storage bags and percent weight loss inside bags measured for two time intervals, 15 and 30 d; time was taken as a within subject factor for a repeated measure ANOVA. In order to distinguish between feeding habits of two species, per grain damage by both species in different wheat varieties was calculated in Microsoft Excel Sheet (Microsoft Corporation Version 2019). For the second experiment, data were analyzed by an independent sample *t*-test at 95% CI. In the second experiment, two bag types, namely hermetic and woven plastic bags, were treatment variables, and holes and penetrations caused by *T. granarium* or *R. dominica* were dependent variables. In the third experiment, data were analyzed by 1-way ANOVA in which mortality of both insects due to phosphine fumigation was the dependent variable, and the three bag types were independent variables. In both the first and third experiments (with three levels of treatments), means were separated post hoc by the HSD test at 5 % level of probability.

## 3. Results

### 3.1. Choice Test (between Invasions and Penetrations)

#### 3.1.1. Effect of Bag Types, Wheat Varieties, or Their Interactions; and Time Period on Live Insects and Percent Weight Loss in Wheat Caused by *R. dominica*

Live insects were recorded only in jute bags while no insects were recorded in woven plastic bags or hermetic plastic bags; corresponding weight loss was recorded in jute bags only compared with woven plastic bags and hermetic plastic bags (F 2, 18: 27.916; *p*: <0.001). Similarly, there was significant effect of varieties on live insects and their percent weight loss in wheat (F 2, 18: 4.330; *p*: 0.029).

Among the varieties, more insects after 15 d were recorded in variety Dilkash (4.67), followed by in Bakhar star (0.66), and none in Akbar (0.00). More weight loss occurred in variety Dilkash (0.11%), followed by Bakhar star (0.03%), and none in Akbar (0.0%).

**Figure 1:**
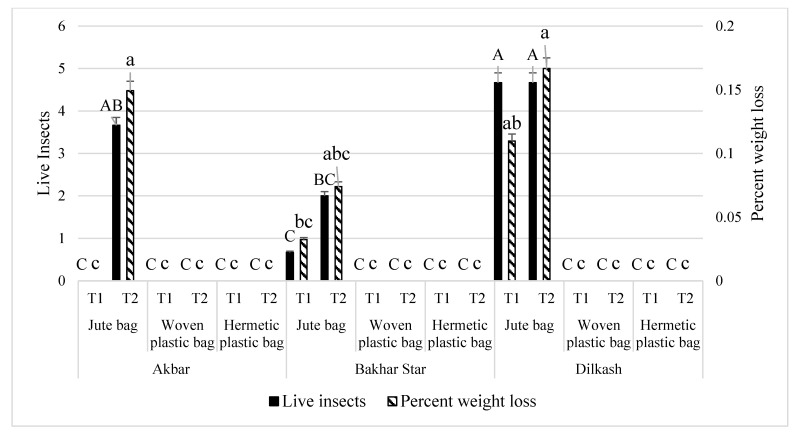
Live insects and percent weight loss in wheat caused by *R. dominica* due to interaction between bag types and wheat varieties. Bars (means) with same (uppercase for live insects and lower case for percent weight loss) letters do not differ significantly across treatments, according to the Tukey HSD test at *p* < 0.05; N: 3 (replicates for bag types and varieties); T1: 15 d, T2: 30 d for repeated measures (live insects and percent weight loss).

After 30 d, more insects were recorded in variety Dilkash (4.67), followed by in Akbar (3.67), and minimum in Bakhar star (2.00). More weight loss occurred in variety Dilkash (0.17%), followed by in Akbar (0.15%), and minimum in Bakhar star (0.074%) (Figure 1).

#### 3.1.2. Effect of Bag Types, Wheat Varieties, or Their Interactions; and Time Period on Live Insects and Percent Weight Loss in Wheat Caused by *T. granarium*

Live insects were recorded only in jute bags while no insects were recorded in woven plastic bags and hermetic plastic bags; corresponding weight loss was recorded in jute bags only compared with woven plastic bags and hermetic plastic bags (F 2, 18: 317.20; *p*: < 0.001). Similarly, there was significant effect of varieties on live insects and their percent weight loss in wheat (F 2, 18: 4.713; *p*: 0.023).

Among the varieties, more insects after 15 d were recorded in variety Bakhar star (34.00), followed by Dilkash (32.00), and minimum were in Akbar (29.67). More weight loss occurred in variety Bakhar star (2.41%), followed by in Akbar (1.28%), and minimum in Dilkash (0.95%).

**Figure 2:**
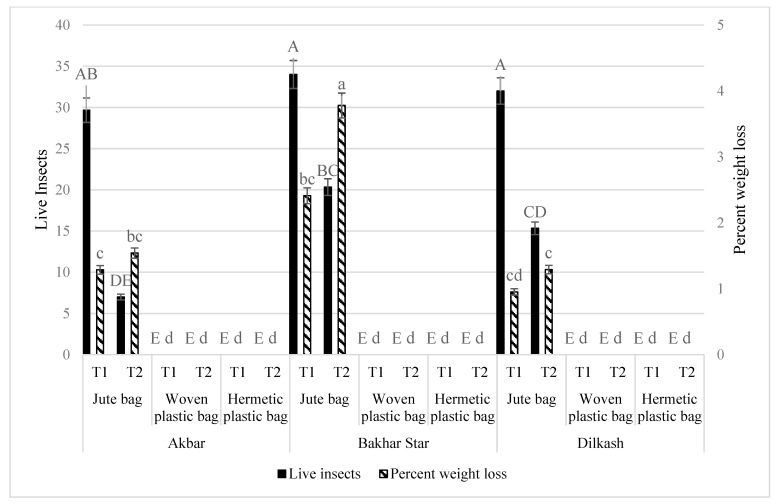
Live insects and percent weight loss in wheat caused by *T. granarium* due to interaction between bag types and wheat varieties. Bars (means) with same (uppercase for live insects and lower case for percent weight loss) letters do not differ significantly across treatments, according to the Tukey HSD test at *p* < 0.05; N: 3 (replicates for bag types and varieties); T1: 15 d, T2: 30 d for repeated measures (live insects and percent weight loss).

After 30 d, more insects were recorded in variety Bakhar star (20.33), followed by Dilkash (15.33), and minimum in Akbar (7.00). More weight loss occurred in variety Bakhar star (3.78%), followed by in Akbar (1.54), and minimum in Dilkash (1.29%) (Figure 2).

#### 3.1.3. Per Grain Damage by *T. granarium and R. dominica*

In order to understand the feeding habits of both insects, damage per grain by *T. granarium* and *R. dominica* in all varieties was determined by measuring weight of all damaged grains by both insects, and their average weight was determined for both insects in three varieties (Table S1: Comparison of per grain damage by *T. granarium* and *R. dominica.*). In all varieties, the average weight of a single damaged grain by *T. granarium* ranged from (28.645 mg) in Bakhar star to (31.596 mg) in Dilkash and (37.722) in Akbar. For *R. dominica*, the average weight of a damaged grain ranged from (19.5) in Akbar to (21.111) in Dilkash and (24.167) in Bakhar star. Therefore, in general, *R. dominica* caused more damage per grain than *T. granarium*.

### 3.2. No Choice Test (for Penetrations Only)

#### 3.2.1. Effect of Bag Types on Damage (Holes and Penetrations) in Bags by *T. granarium* and *R. dominica*

**Figure 3:**
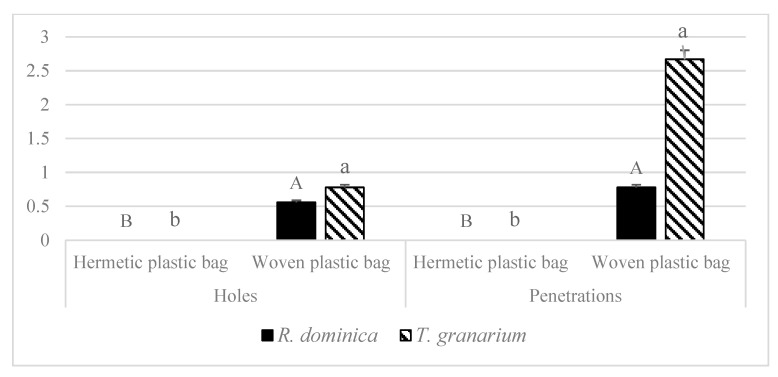
Effect of bag types on damage (holes and penetrations) in bags by *T. granarium* and *R. dominica*. Bars (means) with different (uppercase for *R. dominica* and lower case for *T. granarium*) letters differed significantly across treatments, according to the Tukey HSD test at *p* < 0.05. N: 9 (replicates for bag types); ANOVA parameters for (*R. dominica*) holes were: *t* = −2.294, *p*: 0.025, for penetrations: *t*: −2.401, *p*: 0.022, for (*T. granarium*) holes were: *t* = −2.800, *p*: 0.012, for penetrations: *t*: −2.921, *p*: 0.010, in all cases df = 8.000.

Results of the second experiment showed *R. dominica* created holes (0.556) only in woven plastic bags but not in hermetic plastic bags. Similarly, *R. dominica* made penetrations (0.778) in woven plastic bags only but not in hermetic plastic bags (Figure 3; *p* < 0.05). *T. granarium* species also created holes (0.778) in woven plastic bags but not in hermetic plastic bags. Similarly, penetration (2.667) by *T. granarium* were recorded in woven plastic bags but not in hermetic plastic bags (Figure 3; *p* < 0.05).

#### 3.2.2. Effect of Bag Types on Phosphine Fumigation Efficacy for *R. dominica* and *T. granarium*

**Figure 4:**
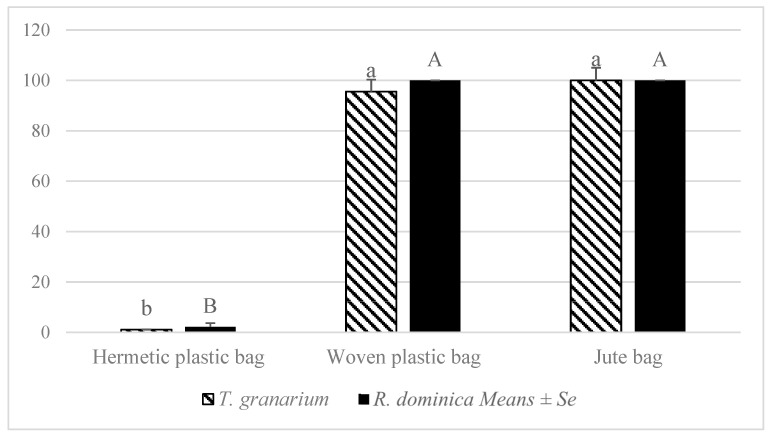
Mortality of *T. granarium* and *R. dominica* due to phosphine fumigation as affected by bag types. Bars (means) with different (uppercase for *R. dominica* and lower case for *T. granarium*) letter differed significantly across treatments, according to the Tukey HSD test at *p* < 0.05. N: 9 (replicates for bag types); ANOVA parameters for *R. dominica* were: F = 1318.435, *p*: <0.001, for *T. granarium*: F: −2.921, *p*: 0.010, in both cases df = 8.000.

Mortality of *T. granarium* was recorded as maximum (100%) in jute bags followed by woven plastic bags (95%), while the least mortality was recorded in hermetic plastic bags (1%). Mortality of *R. dominica* was recorded as maximum in both jute bags and woven plastic bags (100%), while least mortality (2.22%) was recorded in hermetic plastic bags (*p*: <0.05) (Figure 4).

## 4. Discussion

The effect of three types of storage bags, namely jute bag, woven plastic bag, and hermetic plastic bag, with regard to insect infestation and damage to wheat grains inside showed that, in separate experiments for both species, maximum numbers of *R. dominica* as well as *T. granarium* and their damages in terms of percent weight loss were recorded in jute bags; however, no insects caused damage to the other two bags, namely woven plastic bags and hermetic plastic bags, and the grains inside these bags remained safe. This is because jute bags have openings in them, particularly after these are filled with grains; due to that feature, both insects entered jute bags, while no insects damaged (created holes) or entered through holes in woven plastic bags or hermetic plastic bags (penetrations) in the presence of jute bags. Storage insects have been classified as invaders (which can enter packaging only through existing holes and cannot create holes by themselves) and penetrators (which can create holes in packaging and enter through those holes); yet, it has been stated that both penetrators and invaders will exploit package flaws or other existing openings in order to reach a food product [9]. Although both *R. dominica* and *T. granarium* have been categorized as penetrators of packaging, due to their opportunistic behavior of availing themselves of existing openings, both preferred to enter jute bags.

Inside bags were grains of three wheat varieties and among the three wheat varieties, more *T. granarium* and more percent weight loss were recorded in variety Bakhar star, followed by Dilkash, and the least numbers of insects and percent weight loss occurred in Akbar. For *R. dominica*, more live insects and more weight loss occurred in Dilkash, followed by Akbar in descending order, and the least insects and weight loss occurred in Bakhar star. These results can be compared with study findings of [19], which reported relative resistance of different wheat varieties against the infestation caused by *R. dominica* and *T. granarium.* Their findings revealed that weight loss percentage and grain damage was mostly reliant on the development of progeny and also reliant on the varietal preference of the pests.

Effect of time period showed more *R. dominica* and more weight loss occurred after the 30-d period than after 15 d. On the other hand, more *T. granarium* larvae were recorded after 15 d than after 30 d even although more weight loss occurred after 30 d than after 15 d for this pest. These results are in agreement with study reports of [20], which stated that insect numbers and weight loss in stored grains increased with increase in time period. Weight loss and numbers of *R. dominica* increased with time; for *T. granarium*, weight loss increased with time, but their numbers were recorded less after a longer time period than after a shorter time period. This could be because these insects have hairs on their bodies, are positively thigmotactic, and are easily picked by fibers of jute [21]; therefore, some of the larvae might be concealed within the fibers of jute bags which led to the mistake in the counting of *T. granarium*.

In order to show the feeding habits of insects, per grain damage was calculated. Results showed *R. dominica* caused more damage per grain than *T. granarium*. This is in agreement with our recent study findings [22] which showed *R. dominica* caused deep internal tunneling in wheat grains compared with *T. granarium*, which, on the other hand, caused deep open irregular cavities in grains. For this reason, our current results showed weight of a damaged grain is less for *R. dominica* than for *T. granarium*. For varieties, damage per grain by *T. granarium* was greater: Bakhar star > Dilkash > Akbar. For *R. dominica*, it caused more damage per grain in varieties: Akbar > Dilkash > Bakhar star. This is also somewhat in line with the overall weight loss in varieties caused by these pests.

The second experiment (no choice test for penetrations only) involved testing of bags for damage and insect penetration in bags which suffered no attack or penetration by insects in the first experiment, namely woven plastic bag and hermetic plastic bags. The results of this experiment showed both insect species made holes and penetrated into woven plastic bags (although these are very tight and thick), but no insects created holes and entered in hermetic plastic bags. It shows that the relative rough surface and woven nature of plastic in woven plastic bags, compared with hermetic plastic bags which have smooth surface, paved the way for insects to create holes and enter these bags. In addition, hermetic bags plastic is sufficiently thick, which made them less prone to insect attack from outside. This is in agreement with earlier studies which demanded laminations on woven plastic bags to avoid holes and penetrations by different insect pests [23].

Fumigation results showed maximum mortality of both species in jute and woven plastic bags but least mortality in hermetic plastic bags. Maximum mortality in both jute and woven plastic bags due to phosphine fumigation make them superior over hermetic bag, which, on the other hand, showed the least mortality of both insects. Least mortality (<3%) in hermetic plastic bags for both species due to fumigation applied from outside revealed the hermetic plastic bags’ film impermeability to fumigant gas. Although these proved resistant to external insect infestation, impermeability of their plastic film to fumigant gas needs attention.

It is important to note that insects in hermetic bags also remained unaffected due to their own hermetic mode of action during this minimal period of fumigation testing ≈ 1 d both in treatment (with phosphine) as well as in control treatment (without phosphine).

In summary, bags made of jute resulted in early invasions by insects, and grains suffered significant weight loss in wheat. In the absence of jute bags with existing openings, both penetrator insects created holes in woven plastic bags and caused significant weight loss in wheat in these bags. Hermetic plastic bags proved resistant to external infestation caused by *R. dominica* and *T. granarium*. However, when these insects were packed with grains in bags, only jute and woven plastic bags became permeable to external fumigant gas and resulted in maximum mortality of pests, but hermetic plastic bags were impermeable to fumigant gas applied from outside and resulted in no mortality due to fumigation from outside.

## 5. Conclusions

These results suggest alterations in creation of all three types of bags if any of these bags are to be used successfully—either against external infestations or when infestation is already present in the grains. Therefore, this study recommends use of internal plastic liners for both jute and woven plastic bags to resist external insect infestation, particularly with polymers which are not only resistant to insect infestations, but also are permeable to fumigant gases; for example, polypropylene at 0.04 mm thickness can be used to kill internal pests by fumigation from outside. Hermetic plastic bags, being impermeable to external fumigant gas, should be improved regarding their hermetic technology mode of action to generate rapid killing of the pests; preferably, this should be as early as fumigation period ranging from one to several days to restrict damage by these pests because extended survival of pests in these bags will also result in significant damage to grains.

## Data Availability

The data presented in this study are available in Supplementary Materials.

## Supplementary Materials

The following are available online at https://www.mdpi.com/article/10.3390/insects13100955/s1, Table S1: Comparison of per grain damage by *T. granarium* and *R. dominica.*
