# Supplementary material for: Laboratory Evaluation of Storage Bags for Infestations in Wheat Caused by Rhyzopertha dominica F. (Coleoptera: Bostrichidae) and Trogoderma granarium Everts (Coleoptera: Dermestidae) and Their Control Using Phosphine Fumigation
Source: Insects. 2022 Oct 19;13(10):955. doi: 10.3390/insects13100955 (PMC9603926; doi:10.3390/insects13100955)
Supplement: Supplementary file 1 [file insects-13-00955-s001.zip › insects-1931797-supplementary.pdf]

Table S1. Comparison of per grain damage by *T. granarium* and *R. dominica.*

Variety	Weight (mg) of a grain damaged by *T. granarium*		Weight (mg) of a grain damaged by *R. dominica*	
	**15 days**	**30 days**	**15 days**	**30 days**
Akbar	32.143	41.25	-	15
Akbar	35.455	34.194	-	24
Akbar	29.375	-	-	-
**Mean**	**32.324**	**37.722**	**-**	**19.5**
Dilkash	32.5	36.667	24	22.222
Dilkash	31.176	26.875	30	20
Dilkash	31.111	28.462	21.667	-
**Mean**	**31.596**	**31.596**	**25.222**	**21.111**
Bakhar star	28.75	28.889	-	28.333
Bakhar star	26.875	29.167	26.667	20
Bakhar star	26.552	27.879	-	-
**Mean**	**27.392**	**28.645**	**26.667**	**24.167**

(-) symbol represents no grains were found damaged in a particular replication in the experimental setup; bold values are means of weights of individual damaged grains by two different species.
